# Effectiveness of Melatonin‐Containing Foods on Promoting Sleep: A Scoping Review

**DOI:** 10.1002/fsn3.71823

**Published:** 2026-04-27

**Authors:** Tanzeela Nisar, Andrea Fuller, Prajwal Gyawali, Stephen Bird

**Affiliations:** ^1^ School of Health and Medical Sciences, University of Southern Queensland Ipswich Queensland Australia

**Keywords:** actigraphy, dietary melatonin, melatonin‐rich foods, Pittsburgh Sleep Quality Index, sleep, tart cherry

## Abstract

Melatonin plays a crucial role in managing the body's sleep–wake cycle, and its natural occurrence in certain foods has generated interest in dietary interventions to improve sleep. This scoping review explores the impact of consuming foods that naturally contain melatonin on various sleep quality parameters. The literature was obtained through four major databases: PubMed, Scopus, CINAHL, and Web of Science using PRISMA guidelines. A total of 19 articles were included, comprising 14 interventional trials and 5 cross‐sectional studies. Key data were extracted on melatonin sources, dosages, sleep assessment methods, and reported effects on sleep. The quality of articles included was evaluated using the Joanna Briggs Institute (JBI) appraisal tools. The findings of interventional studies suggested that melatonin‐rich foods, including Montmorency tart cherries, Jerte Valley cherries, tomatoes, and kiwifruit, may contribute to improvements in sleep quality, duration, and efficiency, particularly among individuals experiencing sleep disturbances; however, the findings remain heterogeneous. Observational studies also suggested possible associations between higher intake of melatonin‐rich foods and favorable sleep‐related outcomes, including sleep quality, circadian rhythm regulation, and reduced social jetlag, although findings were heterogeneous across populations and study designs. However, studies were limited by variability in population, timing of consumption, melatonin dosage, intervention duration, and sleep assessment tools. Most of the studies focused on a narrow range of foods, despite the increased availability of other melatonin‐rich options that remain underexplored. Future research should prioritize the use of objective tools to assess sleep and investigate a more diverse range of dietary melatonin sources to gain a better understanding of their potential role in sleep regulation.

## Graphical abstract

Effectiveness of melatonin‐containing foods promoting sleep: A scoping review.

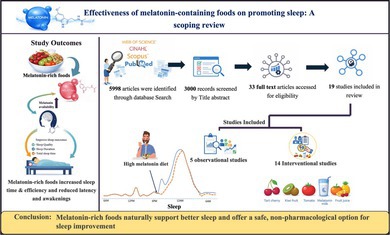

## 1 Introduction

Sleep is a fundamental biological process, primarily regulated by neurobiological pathways, and is crucial for maintaining overall health and well‐being (Halson 2008). Sleep helps the body to replenish its energy reserves, reinforce immune defenses, and improve cognitive functioning (Samuels 2008). Research has highlighted that sleep insufficiency related to duration, continuity, depth, and free from disruptions, is crucial for supporting optimal health (Cook and Charest 2023) ensuring effective learning and memory consolidation (Rasch and Born 2013), emotional stability (Vandekerckhove and Wang 2018) and hormonal regulation (Kim et al. 2015). Inadequate sleep has been identified as a risk factor for several diseases including cardiovascular diseases (Cook and Charest 2023), hypertension (Calhoun and Harding 2010), vascular complications (Kohansieh and Makaryus 2015), metabolic dysfunction (Knutson et al. 2007), and neurocognitive decline (Cappuccio et al. 2010).

Both the duration and quality of sleep can be influenced via pharmacological or non‐pharmacological interventions (Irish et al. 2015). A variety of approaches have been explored to address sleep disturbances, with previous interventions primarily focused on behavioral and educational strategies, such as cognitive‐behavioral therapy and sleep hygiene education (Gadam et al. 2023). Over time, these strategies have expanded to include dietary modifications, environmental changes, and other innovative lifestyle‐based approaches, offering a more comprehensive range of solutions for improving sleep quality (St‐Onge et al. 2016; Rossman 2019). Research has highlighted that the regulation of the sleep–wake cycle involves the coordinated action of several neurotransmitters, including melatonin, orexin, melanin‐concentrating hormone, 5‐HT, gamma‐aminobutyric acid (GABA), cholinergic, noradrenaline, and histamine (Oh et al. 2019). Among them, melatonin (N‐acetyl‐5‐methoxytryptamine) is reported as a pivotal hormone secreted by the pineal gland of the brain and plays a key role in regulating the sleep–wake cycle by a sleep‐promoting effect (Zisapel 2018).

Melatonin is known as a biological modulator of various physiological processes including mood regulation, sleep initiation, retinal function, synchronization of circadian rhythms, and modulation of the immune system (Ahmad et al. 2023). Melatonin secretion can be adversely affected by factors such as disrupted sleep routines, prolonged exposure to artificial light, chronic stress, underlying health conditions, and the use of certain medications (Hardeland 2012). As a response to this reduction, it may be required to use exogenous melatonin or other evidence‐based interventions to effectively support and optimize sleep health. Supplementation with exogenous melatonin improves the restorative quality of sleep and helps in the regulation of the circadian rhythm by lowering core body temperature and increasing sleep onset likelihood (Costello et al. 2014; Nobari et al. 2023).

Melatonin supplementation has been shown to be effective in enhancing sleep onset, duration, and overall quality in children, adolescents, older adults, and postmenopausal women (Mantle et al. 2020; Amstrup et al. 2015; Xie et al. 2017). However, despite its efficacy, there are concerns regarding the potential side effects of its long‐term use, including headaches, daytime sleepiness, and disrupted sleep architecture (Xie et al. 2017). Synthetic melatonin has been associated with gastrointestinal issues, including nausea, abdominal cramps, and diarrhea (Buscemi et al. 2005). Melatonin, derived from food sources, presents a potentially safer approach compared to pharmacological melatonin for enhancing sleep quality (Sae‐Teaw et al. 2013). It holds many advantages over supplementation, like each food has its unique composition of nutrients and bioactive compounds which work synergistically to support health. It is difficult to achieve the same natural equilibrium using supplements (Meng et al. 2017). Secondly, the bioavailability of nutrients derived from whole food is often higher, which ensures that the body can absorb and utilize them more efficiently. Natural melatonin is also less likely to upset the natural balance of the body or cause adverse effects (Muñoz‐Jurado and Escribano 2024). In addition, it will also supply anti‐inflammatory and antioxidant properties that benefit overall health and recovery process (Reiter et al. 2005). It can also help athletes in synchronizing their circadian rhythms for improved performance, making it a safer and more comprehensive option than synthetic interventions (Meng et al. 2017).

For decades, melatonin was regarded solely as an animal neurohormone (Salehi et al. 2019). However, in 1995, its occurrence in higher plants was first identified (Xie et al. 2017). In recent years, melatonin has been recognized as being widely present in a variety of dietary sources derived from both animals and plants with notable variability in concentration depending on the type of food, variety, processing conditions, and time of harvest or collection (Arnao et al. 2023; Zhao et al. 2019). Animal‐based foods such as milk, eggs, and fish have been reported to contain measurable amounts of melatonin (Tan et al. 2014; Milagres et al. 2014). Plant‐based sources, often exhibit much higher melatonin levels (Salehi et al. 2019), particularly in nuts such as pistachios (233.00 ng/g), walnuts (3.5 ng/g), and almonds (39 ng/g) (Muñoz‐Jurado and Escribano 2024; Verde et al. 2022; Oladi et al. 2014). Certain cereals, including wheat, barley, and oats contain relatively high levels of melatonin, with concentrations of 14.9, 6.0, and 7.7 ng/g, respectively (Meng et al. 2017). In terms of fruits, tart cherries, contain 13–15 ng/g of melatonin, goji berries (530 ng/g), cranberries (96 μg/g), grapes (9.3 ng/g) and strawberries (11.26 ng/g) (Meng et al. 2017; Mannino et al. 2021; Nawaz et al. 2015).

Extensive data on melatonin concentrations across a wide range of food sources have been published. Readers are directed to the works by Muñoz‐Jurado and Escribano (2024) and Nawaz et al. (2015) which remain among the most comprehensive on this topic. Meng et al. (2017) demonstrated that melatonin consumed through food is well absorbed and enters the blood circulation, potentially exerting physiological effects through receptor‐mediated or non‐receptor‐mediated processes. It is able to pass the blood–brain barrier (Ng et al. 2017). Emerging evidence from human studies also suggests that the consumption of melatonin‐containing foods may influence circulating melatonin levels in humans. An integrative review by Pereira et al. (2022) reported increase in serum melatonin or urinary 6‐sulfatoxymelatonin following the intake of melatonin rich foods such as cherries, grapes, bananas, pineapples, and certain vegetables (Pereira et al. 2022). Importantly, the authors highlighted that observed increases may not be attributable solely to melatonin content per se, but also to melatonin precursors such as tryptophan and serotonin, as well as antioxidant components present in these foods. Consumption of melatonin‐rich foods has been reported to significantly elevate its circulating levels in humans, with positive effects on insomnia and other various human pathologies (Bravo et al. 2013; Oba et al. 2008; Salehi et al. 2019).

However, despite increasing interest in dietary sources of melatonin, evidence regarding their effects on sleep‐related outcomes remains limited and inconsistent. Previous literature has largely examined the physiological impact of dietary melatonin intake in humans, with primary emphasis on changes in circulating melatonin or its metabolites. But this review is extending the literature by particularly focusing on functional sleep outcomes of dietary melatonin.

The present scoping review was therefore undertaken to systematically map and evaluate evidence from human interventional and observational studies on the effects of melatonin‐containing foods on different sleep outcomes, including sleep quality, sleep duration, sleep efficiency, and related sleep parameters. By incorporating recent evidence and emphasizing validated subjective and objective sleep measures across diverse populations, this review aims to clarify the current state of evidence, identify sources of heterogeneity, and highlight gaps requiring further investigation.

Accordingly, the main objectives of this scoping review were to: (1) evaluate the effects of melatonin‐containing foods on sleep‐related outcomes in humans; (2) describe the characteristics of the dietary interventions across included studies, including the specific melatonin‐rich foods used, the reported melatonin concentration or estimated dose (where available), the populations targeted, and the duration of intervention; (3) summarize the sleep assessment methods used across studies (subjective measures vs. objective measures), alongside biochemical indices of melatonin exposure (e.g., urinary melatonin and 6‐sulfatoxymelatonin [aMT6s]).

## 2 Methods

### 2.1 Protocol and Registration

This scoping review was conducted and reported in accordance with the PRISMA‐ScR (Preferred Reporting Items for Systematic Reviews and Meta‐Analyses Extension for Scoping Reviews) guidelines (Page et al. 2021, Tricco et al. 2018). The protocol of review was registered in the international prospective register of systematic reviews PROSPERO (#CRD42024589287) available at https://www.crd.york.ac.uk/prospero/display_record.php?ID=CRD42024589287https://www.crd.york.ac.uk/prospero/display_record.php?ID=CRD42024589287.

### 2.2 Literature Search

The systematic literature search was conducted through PubMed, Scopus, CINAHL, and Web of Science from the earliest available date until May 2025. The search was conducted using keywords connected by Boolean connectors. The electronic databases mentioned above were searched using a combination of keywords and Medical Subject Headings (MeSH) terms for the following search terms: “diet” or “dietary” or “food” or “nutrition” or “natural” and “melatonin” or “melatonin‐rich” or “phyto‐melatonin” and “sleep” or “insomnia” or “dyssomnia” or “circadian” or “jet lag” or “shift workers”. The search strategy was restricted to studies published in English, excluding manuscripts published in other languages. Additionally, the references of included manuscripts were also searched to prevent missing any relevant articles. Furthermore, to ensure a comprehensive search, the first 10 pages of Google Scholar results were also screened for additional eligible studies. The search query and filters used for each database search are detailed in Table S1.

### 2.3 Study Selection and Eligibility Criteria

Eligibility crsiteria for study selection were guided by the following components identified using the PICOS model (Table 1).

**TABLE 1:** PICOS criteria for inclusion in the review.

	Inclusion	Exclusion
Population “P”	Human studies with no age limit	Animal Studies
Intervention “I”	Intake of natural melatonin‐containing foods	Participants receiving any type of melatonin supplements or pharmacological sleeping interventions
Comparison “C”	For interventional study, must include either a placebo or control group or for observational studies, examining melatonin‐rich food intake and its association with sleep outcomes	Studies without experimental conditions, or with other nutritional supplements
Outcomes “O”	Provide quantitative or qualitative data on sleep outcomes Studies measured sleep quality either through a subjective approach i.e., validated questionnaires (e.g., PSQI) or objective methods (e.g., polysomnography)	No sleep data provided
Study design “S”	Human intervention studies, randomized controlled trials, and/or randomized controlled crossover trials Observational studies and studies that used a targeted analytical approach	Case reports, poster abstracts, editorial, and review articles

### 2.4 Study Screening Process

After completing the database search, all duplicate records were identified and removed. The remaining articles were then transferred into the JBI SUMARI platform for screening and review. In the screening process, two reviewers (T.N. and A.F.) independently reviewed titles and abstracts of papers obtained from all databases. All the conflicts that arose from the title and abstract screening were resolved by the third reviewer (P.G.). In a subsequent, full‐text screening phase, the same reviewers thoroughly revised the full texts and checked whether the manuscript fulfilled all the inclusion criteria. A third reviewer (P.G.) reviewed and resolved any discrepancies.

### 2.5 Data Extraction

In the data extraction phase, two authors (T.N. and A.F.) independently extracted data using the JBI SUMARI software. The following data from the full text of selected studies were extracted: first author's name, year of publication, study location, study duration, demographics of participants, study design, number of participants in each group, type, and dosage, and way of intervention and sleep outcomes. Additionally, data on measures of acceptability and feasibility of the interventions, any reported barriers to implementation, and study limitations were also collected where available. The included studies were categorized based on the design into interventional studies (Table 2) and observational studies (Table 3).

**TABLE 2:** Characteristics of interventional studies.

Studies	Year & location of study	Study population	Age	Study duration	Study design	Melatonin source	Results	Sleep measurement tools
Valtonen et al. (2005)	2005, Finland	Study 1: 70 dementia patients Study 2: 81 elderly subjects	Study 1: 81 ± 9 years Study 2: 82.8 ± 8.1 years	Study 1: 8 weeks Study 2: 8 weeks Washout period: 1 week	Double‐blind, placebo‐controlled, crossover studies	Melatonin‐rich Night‐Time Milk Melatonin dose: (10–40 ng/L melatonin)	Study 1: No significant difference in sleep quality Study 2: ↑ Sleep quality ↑ Morning activity ↑ Evening activity during the night milk period.	Study 1: Subjective sleep quality was monitored by trained nurses Study 2: Subjective sleep quality was monitored by trained nurses
Pigeon et al. (2010)	2010, USA	15 older adults with insomnia	71.6 ± 5.4 years	8 weeks (participants received each treatment for 2 weeks) Washout period: 2‐week	Randomized, double‐blind, placebo‐controlled, crossover trial	8 oz. of Montmorency tart cherry juice blended with apple juice twice daily	↓ Sleep latency (SL) ↓ Wake after sleep onset (WASO) ↑ Total sleep time (TST) ↑ Sleep efficiency (SE) ↓ Insomnia severity	Daily Sleep Diaries Insomnia Severity Index (ISI)
Garrido et al. (2010)	2010, Spain	12 participants (6 middle‐aged, 6 elderly)	Group I Middle‐aged group: 35–55 years, Group II: Elderly group: 65–85 years	3‐day trials for different cultivars of cherries Washout period: 1‐week washout periods between each 3‐day intervention	Prospective, crossover design	200 g of cherries twice daily for each of the seven cultivars of Jerte Valley cherries	↑ Actual sleep time (*p* < 0.05) in 6 out of 7 cultivars ↓ Sleep latency (*p* < 0.05) in 3 out of 7 cultivars ↓ Number of awakenings (*p* < 0.05) in 2 out of 7 cultivars ↓ Total nocturnal activity (*p* < 0.05) in 5 out of 7 cultivars ↑ Sleep efficiency (*p* < 0.05) in 1 out of 7 cultivars Level of Sulfatoxymelatonin (aMT6‐s) (*p* < 0.05) ↑in all 7 cultivars	Actigraphy Urinary 6‐Sulfatoxymelatonin (aMT6‐s) Levels
Howatson et al. (2012)	2012, UK	20 Healthy adults 10 M 10F	18–40 years	7‐day intervention period Washout period: 14 days	Randomized, double‐blind, placebo‐controlled, crossover design	30 mL Montmorency tart cherry juice twice daily Placebo: Mixed fruit cordial, no melatonin or athocyanins Melatonin dose: 1420 ng/30 mL serving or ~85,200 ng Day 1	↑ Total sleep time (TST) ↑ Sleep efficiency (SE) ↑ Time in bed (TIB) ↓ Napping time No significant changes in sleep onset latency (SOL) No significant changes in wake after sleep onset (WASO) ↑ Urinary 6‐sulphatoxymelatonin, aMT6s	Actigraphy Subjective sleep questionnaires Urinary 6‐sulfatoxymelatonin (aMT6‐s) levels
Garrido et al. (2013)	2013, Spain	30 participants (10 young, 10 middle‐aged, 10 elderly)	Young group = 20–30 years Middle‐aged Group = 35–55 years Elderly group = 65–85 years	Two 5‐day treatment periods Washout period: 1‐week	A Blind, placebo‐controlled, randomized, crossover design	125 mL of Jerte Valley Cherry Product JVCP twice daily Placebo: Kool‐aid cherry‐flavored drink with no active components Melatonin dose: Each dose of JVCP consist of 141 g fresh of cherries of in equal parts of 4 diff Jerte valley cherry cultivars	↑ Sleep efficiency in elderly participants ↑ Actual sleep time in all age groups ↓ Number of awakenings in all age groups ↓ Total nocturnal activity in all age groups ↓ Sleep latency in middle‐aged and elderly participants ↑ Urinary 6‐sulfatoxymelatonin (aMT6‐s) in all age groups	Actigraphy Urinary 6‐sulfatoxymelatonin (aMT6‐s) Serum cytokines (IL‐1β, TNF‐α, IL‐8)
Nødtvedt et al. (2017)	2017, Norway	74 students with insomnia symptoms	Mean 24.3 years	4 weeks	Randomized, controlled trial1	130 g of kiwifruit before bedtime or a pear as control group Melatonin dose: One kiwi contain 24,000 ng/g of melatonin	↑ Sleep quality (*p* = 0.68) ↑ Daytime functioning (*p* = 0.51) Total sleep time (NS) Sleep latency (NS) Wake time after sleep onset (NS) No significant changes in Actigraphy	Actigraphy Sleep Diary PSQI Bergen Insomnia Scale (BIS)
Losso et al. (2018)	2018, USA	8 participants with chronic Insomnia 5 Females 3 Males	≥ 50 years	2‐week treatment periods Washout period: a 2‐week	Randomized, double‐blind, placebo‐controlled crossover study	240 ml of tart cherry juice twice daily placebo (water, fructose, dextrose, lemon powder) Melatonin dose: 135 ng	↑ Total sleep time by 84 min (*p* = 0.0182) ↑ Habitual sleep efficiency (*p* = 0.03) Wake time after sleep onset: NS (Not Significant) Sleep onset latency: NS Number of awakenings: NS Sleep Efficiency ↑ (NS) ↑ Sleep Efficiency	Polysomnography Subjective sleep questionnaires Pittsburgh Sleep Quality Index (PSQI) Insomnia Severity Index (ISI) Epworth Sleepiness Scale (ESS) Beck Depression Inventory II (BDI‐II) State–Trait Anxiety Inventory (STAI)
Yang et al. (2020)	2020, Taiwan	36 Obese postmenopausal women	< 70 years	8 weeks	Open‐label, randomized controlled dietary intervention trial	250 g beefsteak tomatoes before sleep	↑ Sleep quality ↑ Sleep Efficiency ↓ Sleep Latency ↑ Urinary aMT6s Level	Pittsburgh Sleep Quality Index (PSQI) Urinary 6‐sulfatoxymelatonin (aMT6‐s) level
Kim et al. (2020)	2020, South Korea	25 adults 15Females 10 Males	25 ± 0.1	8 weeks of intervention Washout period: 2‐week	Randomized, crossover design	Sleep‐inducing juice (composed of pineapples, frozen cherries, lettuce and oranges)	↑ Sleep Quality (PSQI) significantly (*p* < 0.001) ↑ Sleep Efficiency: significantly (*p* < 0.05) ↑ Total Sleep Time (TST) significantly (*p* < 0.05) ↓ Sleep Latency significantly (*p* < 0.05)	Actigraphy PSQI
Chung et al. (2022)	2022, Korea	19 elite female field hockey players	21.5 years	48 h	Randomized, double‐blind, placebo‐controlled crossover trial	Tart cherry concentrate (Single serving = 200 mL of beverage containing 30 mL of tart cherry concentrate, consumed for five times over 48 h) (30 L tart cherries concentrate = 48 tart cherries)	↑ Total Time in Bed (TTB) (*p* = 0.015) ↓ Wake After Sleep Onset (WASO) (*p* = 0.044) ↑Movement Index (MI) (*p* = 0.031) Serum Melatonin Levels: No significant changes	Actigraphy was used to measure sleep efficiency (SE), total time in bed (TTB), total sleep time (TST), wake after sleep onset (WASO) number of awakenings (NOA) movement index (MI) Sleep Questionnaire ASBQ ASSQ Serum Melatonin level
Hillman et al. (2022)	2022, USA	44 participants	18–44 years	30 days	Randomized, double‐blind, placebo‐controlled, parallel design	240 mL of Montmorency Tart Cherry juice (MTC) or capsules of freeze dried tart cherries (500 mg) twice daily Placebo: colored corn starch	Sleep Quality & Duration: No significant change (NS) between tart cherry and placebo groups Melatonin Levels: No significant change (NS) in serum melatonin	Self‐reported daily survey Serum melatonin levels
Kanon et al. (2023)	2023, New Zealand	24 healthy adults	29 ± 1 years	Single‐night study with three separate interventions	Randomized, single‐blind crossover study	1: Flesh of two fresh green kiwi fruits (200 g), 2: 32 g Freeze‐dried kiwi fruits (flesh + skin) Control: 200 mL of water	↑ Total Time in Bed (TTB) ↓ Wake After Sleep Onset (WASO) ↓ Number of Awakenings (aMT6s) showed no significant changes (NS)	Pittsburgh Sleep Quality Index (PSQI), Stanford Sleepiness Scale (SSS), Leeds Sleep Evaluation Questionnaire (LSEQ) Actigraphy Urinary aMT6s
Doherty et al. (2023)	2023, Ireland and UK	15 elite athletes	23.2 ± 3.9 years	Intervention period of 4 weeks (1 baseline week)	Randomized, controlled trial, Open label trial	2 kiwifruits per day Melatonin dose: Melatonin content (24,000 ng/g)	↑ Total Sleep Time (TST) ↑ Sleep Efficiency (SE%) ↓ Number of Awakenings (NoA) ↓ Wake After Sleep Onset (WASO) ↑ Sleep Quality (PSQI Global Score improved) (*p* = 0.002) ↓ General Stress ↓ Sport Stress	PSQI Consensus Sleep Diary (CSD‐C)
Tucker et al. (2024)	2024, USA	34 adults with overweight/obesity and poor sleep	Mean 32.6 ± 10.7 years (range: 18–50)	14 days per treatment with 10‐day washout	Randomized, double‐blind, placebo‐controlled, crossover trial	500 mg/day Montmorency Tart Cherry (MTC) powder (100%), freeze‐dried, 1 capsule twice/day	No significant changes in TST, deep sleep, or REM sleep between MTC and placebo groups No significant changes in SQS scores ISI and PSQI scores NS between groups (*p* = 0.92 and *p* = 0.51) NS change in Inflammatory markers (TNF‐α, IL‐6, IL‐8, IL‐10, IL‐17A, CRP)	Zmachine Synergy Sleep Monitor (EEG) Fitbit Inspire 3 Pittsburgh Sleep Quality Index (PSQI) Insomnia Severity Index (ISI) Sleep Quality Scale (SQS)

**TABLE 3:** Characteristics of observational studies.

Study name	Year & location of study	Study population	Age	Study duration	Study design	Melatonin source	Results	Sleep measurement tools
Polugrudov et al. (2023)	2023, Russia	83 young healthy adults 57.8% Females 42.2% Males	26.7 ± 6.1 years	4 months in Syktyvkar (Jan to April 2018) 7 months in in Moscow (Oct 2021 to April 2022)	Cross‐sectional study	Melatonin‐containing foods, assessed by a 24‐h dietary recall and food diary for 7 days	Sleep duration: ↑ increase ~1 h with high FMT dinner intake Deep sleep phase: ↑ increase ~0.5 h with high FMT dinner intake Social Jetlag: ↓ decrease 0.9 h with high FMT dinner intake	PSQI The Munich Chronotype Questionnaire (MCTQ) Dreem2 headband
Borisenkov et al. (2023)	2023, Russia	1277 school children & university students	16–25 years	1 year	Cross‐sectional study	Melatonin‐containing foods (FMT), assessed via food frequency questionnaire (FFQ)	Sleep quality: ↑ Improved with increased daily FMT consumption Chronotype: ↑ Early chronotype associated with higher FMT intake Social Jetlag: ↓ Reduced social jetlag with higher FMT intake Depression scores: ↓ Lower depression scores with higher FMT intake	Munich Chronotype Questionnaire (MCTQ) Pittsburgh Sleep Quality Index (PSQI)
Borisenkov et al. (2024)	2024, Russia	557 Older Adults	Average age of 68.9 ± 7.7 years, ranging from 51 to 90 years	Conducted in May and September 2023	Cross‐sectional study	Melatonin‐containing foods (FMT), assessed via food frequency questionnaire (FFQ)	Sleep quality: No significant association with FMT intake Social Jetlag: No significant association with FMT intake Depression Scores: ↓ Lowered (*p* = 0.003)	Munich Chronotype Questionnaire (MCTQ) Pittsburgh Sleep Quality Index (PSQI)
Zhang et al. (2023)	2023, United States (NHANES 2005–2018)	US adults (NHANES dataset, *n* = 29,217)	≥ 20 years	Cross‐sectional study (2005–2018)	Observational study using NHANES survey data	Berries (strawberries, blueberries, raspberries, blackberries, cranberries)	↓ Short Sleep Risk (10%–17% reduction, *p* < 0.05), NS Long Sleep Risk, ↓ Sleep Difficulty with Blackberry Consumption	NHANES Sleep Disorders Questionnaire (self‐reported sleep duration & difficulty)
Borisenkov et al. (2025)	2025, Russia	587 Children with language difficulties	2–12 years	May 2023 to March 2024	Observational Cross‐Sectional Study	Melatonin‐containing foods (FMT) assessed via Food Frequency Questionnaire (FFQ); included cherries, grapes, tomatoes, walnuts, milk, and other natural dietary sources	No significant association with total sleep duration, sleep quality, or latency ↓ Social jetlag (*p* = 0.005) ↑ Earlier chronotype (mid‐sleep phase advanced; *p* = 0.040) ↑ Circadian alignment (less delayed sleep–wake rhythm)	Self‐reported sleep satisfaction & duration Modified Munich ChronoType Questionnaire (MCTQ)

### 2.6 Collecting, Summarizing, and Reporting the Results

The extracted data were tabulated and summarized descriptively. Interventions were categorized based on the type of melatonin‐containing food source, study duration, and follow‐up period. We also explored how melatonin content was measured or reported in each study. Furthermore, we assessed the effectiveness of the interventions in improving sleep outcomes, as well as other factors relevant to potential implementation, including measures of acceptability, feasibility, reported barriers, and study limitations.

### 2.7 Methodological Quality Assessment

Methodologic quality analysis was done by using the Joanna Briggs Institute (JBI) Appraisal checklist for RCTs, quasi‐experimental studies, and cross‐sectional studies (Tables 4, 5, 6) (Munn et al. 2019). The JBI checklist included an assessment of true randomization, concealed allocation, baseline similarity between treatment and control, blinding, potential dropout bias, reliable outcome measurement, and appropriate statistical analysis for RCTs. For cross‐sectional studies and quasi‐experimental studies, it included 8 items, and each needed an answer of Yes, No, Unclear, or Not Applicable. Studies with more than 70% “Yes” responses were classified as high quality, those with 50%–70% as moderate quality, and those with less than 50% as low quality (George et al. 2014).

**TABLE 4:** Methodologic quality of RCT design using the JBI checklist for RCT.

Study	1. Randomization	2. Allocation concealed	3. Baseline similarity	4. Participant blinding	5. Provider blinding	6. Assessor blinding	7. Identical treatment	8. Follow‐up complete	9. Analyzed per group	10. Consistent outcome measurement	11. Reliable outcome measurement	12. Suitable statistical analysis	13. Suitable trial design	Total score %
Valtonen et al. (2005)	Yes	Unclear	Yes	Yes	Unclear	Unclear	Yes	Yes	Yes	Yes	No	Yes	No	**62**
Pigeon et al. (2010)	Yes	Unclear	Yes	Yes	Yes	Unclear	Yes	Yes	Yes	Yes	No	Yes	Yes	**77**
Howatson et al. (2012)	Yes	Yes	Yes	Yes	No	Unclear	Yes	Yes	Yes	Yes	Yes	Yes	Yes	**85**
Garrido et al. (2013)	Yes	Unclear	Unclear	Yes	Unclear	No	Yes	Yes	Yes	Yes	Yes	Yes	Yes	**69**
Nødtvedt et al. (2017)	Yes	Unclear	Yes	Unclear	Unclear	Unclear	Yes	Yes	Yes	Yes	Yes	Yes	Yes	**69**
Losso et al. (2018)	Yes	Unclear	Yes	Yes	Unclear	Yes	Yes	Yes	Yes	Yes	Yes	Yes	Yes	84
Yang et al. (2020)	Yes	Unclear	Yes	Unclear	Unclear	Unclear	Yes	Yes	Yes	Yes	Yes	Yes	Yes	**69**
Kim et al. (2020)	Yes	Unclear	Yes	No	No	Unclear	Yes	Yes	Yes	Yes	Yes	Yes	Yes	**69**
Chung et al. (2022)	Yes	Yes	Yes	Yes	Unclear	Unclear	Yes	Yes	Yes	Yes	Yes	Yes	Yes	**85**
Hillman et al. (2022)	Yes	Unclear	Yes	Yes	Yes	Yes	Yes	Yes	Yes	Yes	Yes	Yes	Yes	**92**
Kanon et al. (2023)	Yes	Unclear	Yes	No	Unclear	Yes	Yes	Yes	Yes	Yes	Yes	Yes	Yes	**76**
Tucker et al. (2024)	Yes	Unclear	Yes	Yes	Unclear	Unclear	Yes	Yes	Yes	Yes	Yes	Yes	Yes	**62**

*Note:* Study quality is defined as follows: a total score greater than 70% as high quality, a score between 50% and 70% as medium quality, and a score less than 50% as low quality. The use of bold formatting in Table 4 is solely for presentation purpose and does not imply any statistical or methodological importance.

**TABLE 5:** Methodologic quality of quasi experimental studies using the JBI checklist.

Study	1. Cause‐effect clearly defined	2. Comparison groups similar	3. Similar treatment in comparisons	4. Control group present	5. Pre & post outcome measurement	6. Follow‐Up complete & analyzed	7. Consistent outcome measurement	8. Reliable outcome measurement	Total score
Garrido et al. (2010)	Yes	No	No	No	Yes	Unclear	Yes	Yes	56%
Doherty et al. (2023)	Yes	No	No	No	Yes	Yes	Yes	Yes	62%

*Note:* Study quality is defined as follows: a total score greater than 70% as high quality, a score between 50% and 70% as medium quality, and a score less than 50% as low quality.

**TABLE 6:** Methodologic quality of cross sectional studies using the JBI checklist.

Study	1. Inclusion criteria defined	2. Study setting described	3. Exposure measured reliably	4. Standard criteria used	5. Confounding factors identified	6. Strategies for confounding	7. Outcome measured reliably	8. Statistical analysis	Total score
Polugrudov et al. (2023)	Yes	Yes	Yes	Yes	Unclear	No	Yes	Yes	81%
Borisenkov et al. (2023)	Yes	Yes	No	Yes	Yes	Yes	Yes	Yes	88%
Borisenkov et al. (2024)	Yes	Yes	No	Yes	Yes	Yes	Yes	Yes	88%
Zhang et al. (2023)	Yes	Yes	No	No	Yes	Yes	No	Yes	62%
Borisenkov et al. (2025)	Yes	Yes	No	No	Yes	Yes	No	Yes	62%

*Note:* Study quality is defined as follows: a total score greater than 70% as high quality, a score between 50% and 70% as medium quality, and a score less than 50% as low quality.

## 3 Results

### 3.1 Literature Search and Study Selection

**FIGURE 1:**
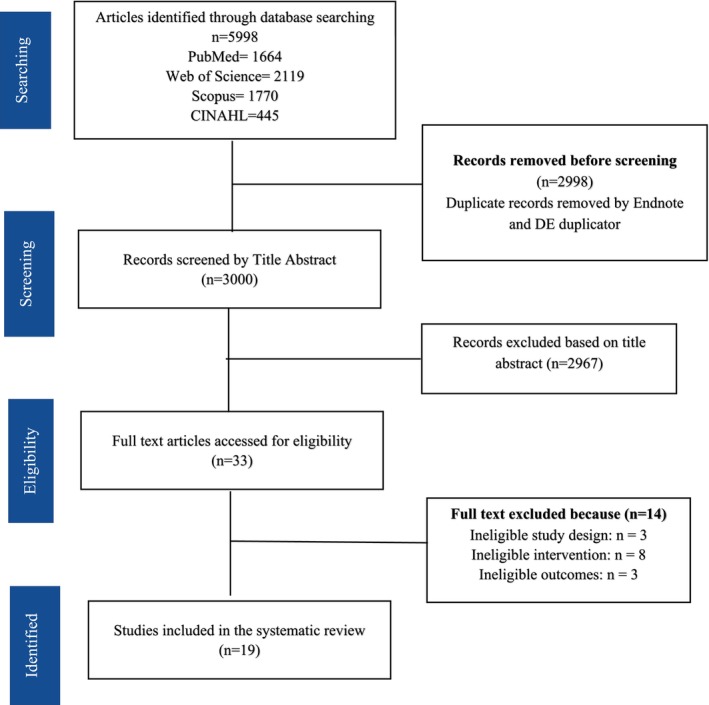
PRISMA flow diagram of the literature search and screening process.

The literature search initially yielded 5998 articles, and after removing duplicates, 3000 abstracts were screened. A total of 33 full‐text articles were originally identified as potentially relevant for this review. However, after following the eligibility criteria, 14 manuscripts were excluded, and 19 articles that met all inclusion criteria were ultimately included in this scoping review (Figure 1).

### 3.2 Overview of Included Studies

The scoping review included 19 studies, involving a total of 32,228 participants, examining the effects of melatonin‐containing food consumption on sleep quality, circadian rhythm, jet lag, and other sleep‐related parameters. Among these studies, 14 were interventional (Valtonen et al. 2005; Pigeon et al. 2010; Garrido et al. 2010, 2013; Howatson et al. 2012; Nødtvedt et al. 2017; Losso et al. 2018; Yang et al. 2020; Kim et al. 2020; Chung et al. 2022; Hillman et al. 2022; Kanon et al. 2023; Doherty et al. 2023; Tucker et al. 2024) and five were observational studies (Borisenkov et al. 2023, 2024, 2025; Polugrudov et al. 2023; Zhang et al. 2023). The studies were geographically diverse, with five studies conducted in the USA (Pigeon et al. 2010; Hillman et al. 2022; Losso et al. 2018; Zhang et al. 2023; Tucker et al. 2024), four in Russia (Borisenkov et al. 2023, 2024, 2025; Polugrudov et al. 2023), two in Spain (Garrido et al. 2010, 2013), one in the UK (Howatson et al. 2012), two in South Korea (Chung et al. 2022; Kim et al. 2020), and each in Taiwan (Yang et al. 2020), Ireland (Doherty et al. 2023), Norway (Nødtvedt et al. 2017), New Zealand (Kanon et al. 2023), and Finland (Valtonen et al. 2005). Sample sizes varied widely, from as few as eight participants (Losso et al. 2018) to as many as 29,217 in the largest observational study (Zhang et al. 2023). Participant ages across the included studies ranged from 16 to 90 years.

#### 3.2.1 Population

The study populations varied widely and included healthy adults, elderly individuals, elite athletes, postmenopausal women, chronic insomnia patients, and individuals with disturbed sleep. Six studies focused on middle‐aged and older adults aged 50 to 85 years (Valtonen et al. 2005; Pigeon et al. 2010; Garrido et al. 2010, 2013; Losso et al. 2018; Borisenkov et al. 2024), while three investigations targeted university students aged 16 to 25 years (Nødtvedt et al. 2017; Borisenkov et al. 2023). Seven studies assessed sleep in young adults (Howatson et al. 2012; Kim et al. 2020; Hillman et al. 2022; Kanon et al. 2023; Polugrudov et al. 2023; Zhang et al. 2023) and two were conducted exclusively among elite athletes (Chung et al. 2022; Doherty et al. 2023). One study focused on obese postmenopausal women (Yang et al. 2020), while one study investigated sleep in children with language difficulties (Borisenkov et al. 2025). Additionally, one study examined sleep outcomes in individuals with obesity (Tucker et al. 2024).

#### 3.2.2 Tools

Various tools were used to measure sleep quality and related parameters across the 19 studies. The Pittsburgh Sleep Quality Index (PSQI) was used in 10 studies (Nødtvedt et al. 2017; Losso et al. 2018; Yang et al. 2020; Kim et al. 2020; Borisenkov et al. 2023, 2024; Kanon et al. 2023; Doherty et al. 2023; Polugrudov et al. 2023; Tucker et al. 2024) to assess subjective sleep quality. Actigraphy was used in seven studies (Garrido et al. 2010, 2013; Howatson et al. 2012; Nødtvedt et al. 2017; Kim et al. 2020; Chung et al. 2022; Kanon et al. 2023) as an objective sleep measurement tool. Polysomnography, considered the gold standard for sleep quality assessment, was utilized in only one study (Losso et al. 2018). Other measurement tools included the Munich Chronotype Questionnaire (MCTQ), which was applied in four studies (Borisenkov et al. 2023, 2024, 2025; Polugrudov et al. 2023) to evaluate chronotype and social jetlag. The Consensus Sleep Diary (CSD‐C) was employed in one study (Doherty et al. 2023), while daily sleep diaries and subjective sleep questionnaires were used in four studies (Valtonen et al. 2005; Pigeon et al. 2010; Nødtvedt et al. 2017; Hillman et al. 2022) for self‐reported sleep patterns. Urinary 6‐Sulfatoxymelatonin (aMT6‐s) levels were analyzed in five studies (Garrido et al. 2010, 2013; Howatson et al. 2012; Yang et al. 2020; Kanon et al. 2023) to assess melatonin metabolite secretion in urine. Additionally, serum melatonin levels were measured in two studies (Hillman et al. 2022; Chung et al. 2022). Psychological and cognitive tools were also included in some studies. The Beck Depression Inventory (BDI‐II) was used in one study (Losso et al. 2018), while the Epworth Sleepiness Scale (ESS) was applied in the same study to assess daytime sleepiness. The Insomnia Severity Index (ISI) was used in three studies (Pigeon et al. 2010; Losso et al. 2018; Tucker et al. 2024) to evaluate insomnia symptoms. Additional subjective sleep measures Stanford Sleepiness Scale (SSS) and the Leeds Sleep Evaluation Questionnaire (LSEQ) were used in one study (Kanon et al. 2023).

### 3.3 Quality Assessment

Nineteen studies are included in the current review, consisting of 12 randomized controlled trials (RCTs), 2 quasi‐experimental studies, and 5 cross‐sectional studies. The methodological quality assessment of these studies is detailed in Tables 4, 5, 6. Among the 12 RCTs, 7 were classified as high‐quality studies (score > 70%), and 5 were of medium quality (score 50%–70%), based on the JBI Checklist for RCTs. The most common methodological shortcomings in high‐quality RCTs were: failure to blind the interventionists (*n* = 8, 67%), inability to conceal allocation (*n* = 7, 58%), and lack of participant blinding (*n* = 5, 42%). The most frequent methodological weaknesses in quasi‐experimental studies were: lack of a control group (*n* = 1, 50%), failure to define cause‐effect relationships (*n* = 2, 100%), inconsistent follow‐up analysis (*n* = 2, 100%), reliance on self‐reported data rather than objective outcome measures (*n* = 2, 100%). Among 5 cross‐sectional studies, 2 studies were of high quality, while 3 were of medium quality. The most common limitations were: exposure measurement based on self‐reports (*n* = 4, 80%), confounding factors not fully addressed (*n* = 3, 60%), lack of standard objective criteria for outcome measurement (*n* = 3, 60%).

### 3.4 Sleep Outcomes From Interventional Studies on Dietary Melatonin Intake

Tart cherry was the most frequently studied melatonin‐rich food source, used in eight studies (Pigeon et al. 2010; Howatson et al. 2012; Losso et al. 2018; Chung et al. 2022; Hillman et al. 2022; Tucker et al. 2024; Garrido et al. 2010, 2013). The potential sleep‐promoting effects of tart cherries were investigated via a mix of study designs, targeting both individuals with sleep disturbances and healthy populations across different age groups. These studies examined various forms of tart cherries, including fresh fruit, juice, concentrate, and powdered preparations. Pigeon et al. conducted a crossover study in chronic insomnia and found that Montmorency tart cherry juice (MTC) (230 mL twice daily) significantly improved total sleep time and sleep efficiency (Pigeon et al. 2010). Similarly, Howatson et al. reported that MTC juice (30 mL twice daily, providing 85.2 μg of melatonin per day) significantly increased urinary melatonin levels and improved sleep efficiency and total sleep time (Howatson et al. 2012). Losso et al. found that MTC juice (240 mL twice daily) significantly increased total sleep time and sleep efficiency in older adults with insomnia, while other sleep parameters (sleep onset, sleep onset latency) showed no significant changes (Losso et al. 2018).

Chung et al. investigated the effects of tart cherry concentrate (30 mL for five times in 48 h) in elite female athletes and reported significant improvements in total time in bed and reduced WASO (Chung et al. 2022). However, serum melatonin levels did not significantly change, which the authors attributed to individual variability in melatonin metabolism, the short half‐life of melatonin, or diurnal fluctuations. The study suggested that polyphenols and antioxidative compounds in tart cherries, rather than only melatonin, may have contributed to the sleep benefits. An additional RCT in healthy adults compared the effects of MTC juice (240 mL twice daily) and powdered tart cherry capsules over a 30‐day period. The study found no significant improvements in sleep duration or serum melatonin levels, suggesting the limited effectiveness of tart cherry in individuals without baseline sleep disturbances (Hillman et al. 2022). In another study, Tucker et al. investigated the effects of MTC powder (500 mg capsules) in adults with obesity (Tucker et al. 2024). No significant improvements were observed in objective or subjective sleep outcomes, serum melatonin levels, or inflammatory biomarkers, suggesting individuals with higher BMI may need higher levels of supplementation to achieve the effects reported in other studies.

Two studies (Garrido et al. 2010, 2013) examined Jerte Valley cherries (JVC) and reported significant improvements in sleep quality. In one study, 200 g of JVC enhanced sleep–wake rhythms and increased urinary aMT6‐s levels in elderly adults (Garrido et al. 2010). Another study found that a JVC product (equivalent to 141 g fresh cherries) improved total sleep time, sleep latency, and reduced wakefulness across various age groups (Garrido et al. 2013). Three included studies examined the effect of consumption of kiwifruit on sleep. One RCT in students with chronic insomnia reported improved self‐reported sleep quality, though actigraphy showed no significant changes (Nødtvedt et al. 2017). A crossover trial found that consumption of 2 dried kiwis improved ease of awakening in poor sleepers, while fresh kiwi improved sleep onset in good sleepers, with no rise in urinary melatonin, suggesting that other nutrient components in the kiwi fruit, rather than melatonin, may be the mechanism driving sleep improvements (Kanon et al. 2023). While in elite athletes, a 4‐week trial with two green kiwifruits nightly improved sleep duration, efficiency, and PSQI scores (Doherty et al. 2023).

A study involving obese postmenopausal women found that consumption of 250 g of beefsteak tomatoes significantly improved sleep quality and led to a 10‐fold increase in urinary 6‐sulfatoxymelatonin levels (Yang et al. 2020). In an additional included study, the effects of a sleep‐inducing juice containing pineapple, frozen cherry, orange, and lettuce was evaluated in adults with disturbed sleep. Consumption of juice for 8 weeks resulted in significant improvements in sleep parameters (C.S. Kim et al. 2020). Valtonen et al. evaluated the effects of milk collected at night‐time, naturally higher in melatonin 0.0855 ng/g, on sleep quality and daytime activity in elderly participants. Despite the higher melatonin content in nighttime milk, the study found no significant improvements in sleep quality. However, some participants showed increased daytime and evening activity, suggesting a possible role in circadian rhythm regulation rather than direct sleep enhancement (Valtonen et al. 2005).

### 3.5 Sleep Outcomes From Cross‐Sectional Studies on Dietary Melatonin Intake

Five cross‐sectional studies have been included in this review that examined the relationship between melatonin‐containing food (FMT) consumption and sleep‐related outcomes in different populations, including young adults, older adults, and the general population. In each study, FMT intake was assessed using a modified food frequency questionnaire (FFQ), which captured details such as frequency, portion size, and timing of food consumption. One study in young adults with social jetlag reported that higher intake of FMT at dinner was associated with an additional hour of total sleep and a 30 min increase in deep sleep duration, alongside a 0.9‐h reduction in social jetlag (Polugrudov et al. 2023). Similarly, among students aged 16–25, greater FMT intake correlated with earlier chronotype, improved sleep quality, reduced social jetlag, and lower depression scores, indicating that FMT consumption may support the alignment of sleep–wake cycles with circadian rhythms (Borisenkov et al. 2023). In contrast, a study in older adults found no direct association between FMT intake and sleep duration or quality, although improved psychoemotional well‐being and life satisfaction were observed (Borisenkov et al. 2024).

Additionally, a study in children with language difficulties found that while higher FMT intake was not associated with sleep duration or efficiency, it was linked to reduced social jetlag and fewer behavioral problems, highlighting the potential role of dietary melatonin in regulating the body's internal clock and improving circadian rhythm alignment in children with language difficulties (Borisenkov et al. 2025). A large‐scale cross‐sectional study examined the relationship between berry consumption as a source of melatonin and sleep outcomes. Dietary intake was assessed through two non‐consecutive 24‐h dietary recalls, and sleep quality was measured by self‐reported sleep duration. The study found that a higher intake of strawberries and blueberries was associated with a 10%–17% lower risk of short sleep (< 7 h) (*p* < 0.05), while blackberry consumption was linked to a 37% lower risk of sleep disturbances. No associations were found with long sleep duration (Zhang et al. 2023).

## 4 Discussion

This scoping review aimed to identify and summarize the existing evidence on the use of natural melatonin‐containing foods as a dietary strategy for improving sleep quality. We provided a comprehensive overview of both interventional and observational studies examining the impact of various melatonin‐containing foods on sleep‐related outcomes across diverse populations. The review highlights a growing interest in non‐pharmacological, food‐based interventions for sleep improvement. The collective evidence suggests that the consumption of melatonin‐containing foods, such as Montmorency tart cherries, Jerte Valley cherries, kiwifruit, tomatoes, or nighttime milk, may offer potential benefits for sleep enhancement. Cross‐sectional studies within the review also identified a positive association between higher dietary intake of melatonin‐containing foods and better sleep outcomes, supporting the potential of dietary strategies in promoting sleep health. However, notable variation was observed in the outcomes of interventional study results, even among trials that utilized the same food source, highlighting inconsistencies in study design or individual participant responses.

Among the various melatonin‐rich fruits, tart cherries, particularly Montmorency and Jerte Valley varieties, are among the most studied for their potential sleep‐promoting properties. According to Feng et al., Montmorency tart cherries (MTC) contain approximately 13–15 ng of melatonin per gram of fruit, making them one of the rich natural sources of melatonin (Feng et al. 2014). Similarly, Jerte Valley cherries have been reported to contain substantial levels of melatonin, serotonin, and tryptophan, along with high concentrations of anthocyanins and phenolic compounds (Garrido et al. 2010, 2013). This review included eight studies that evaluated the effects of tart cherry consumption on sleep outcomes, primarily through juice, concentrate, or cherry‐based products. One study found that daily consumption of tart cherry juice (42,600 ng/30 mL serving or ~85,200 ng/day of melatonin) significantly increased urinary melatonin levels (aMT6s) by 17%, which correlated with improvements in sleep efficiency and total sleep time (Howatson et al. 2012). Similarly, another study (Pigeon et al. 2010) reported that MTC juice significantly improved insomnia severity scores and total sleep time in older adults, suggesting that melatonin absorption contributed to sleep improvements.

However, it is important to consider that melatonin might not be the sole mechanism driving the sleep improvements associated with tart cherry consumption. Losso et al. proposed a distinct mechanism underlying the sleep‐promoting effects of tart cherry juice. In their study, Procyanidin B‐2, a prominent polyphenolic compound in the juice, was shown to inhibit the activity of indoleamine 2, 3‐dioxygenase (IDO), an enzyme involved in tryptophan catabolism. This inhibition led to a reduction in tryptophan degradation, thereby increasing its availability for serotonin and melatonin synthesis. These biochemical changes were supported by lower kynurenine‐to‐tryptophan ratios and reduced prostaglandin E2 levels, indicating that both anti‐inflammatory and serotonergic pathways may contribute to the observed sleep improvements (Losso et al. 2018). They also emphasized that while tart cherry juice provides approximately 85,000 ng of melatonin per day, this amount is substantially lower than pharmacological doses typically used to treat insomnia, which range from 0.5 to 5 mg/day—a 6‐ to 60‐fold higher intake. This observation aligns with the view that melatonin alone is unlikely to be the sole driver of sleep improvements. Therefore, the beneficial effects of tart cherries on sleep are likely the result of a synergistic interaction between melatonin and other bioactive components, including anti‐inflammatory polyphenols and serotonergic compounds, rather than melatonin alone. Chung et al. also observed improved sleep parameters following short‐term tart cherry supplementation in elite female athletes. However, serum melatonin levels did not significantly change in that study, which the authors attributed to individual variability in melatonin metabolism, the short half‐life of melatonin, or diurnal fluctuations (Chung et al. 2022). The study suggested that the sleep‐promoting effects of tart cherries may not be solely attributed to their melatonin content but also to the presence of polyphenols and other antioxidative compounds, which could influence sleep through anti‐inflammatory pathways and modulation of circadian‐regulating neurochemicals (Chung et al. 2022).

Contrasting results were observed in Hillman's study, which found no significant changes in sleep quality, duration, or melatonin levels in healthy adults after consuming tart cherry juice or capsules for 30 days (Hillman et al. 2022). This suggests that tart cherries may be more beneficial for individuals with sleep disturbances than those with normal sleep patterns. The variability in findings highlights the need to consider baseline sleep status, bioavailability of melatonin, and the synergistic effects of other bioactive compounds when evaluating tart cherries as a sleep aid. Similarly, one more study also found no improvements in sleep outcomes or melatonin levels after MTC powder supplementation in adults with overweight or obesity (Tucker et al. 2024). Two studies (Garrido et al. 2010, 2013) examined Jerte Valley cherries (JVC) and reported significant improvements in sleep quality and increased urinary aMT6‐s levels in different age groups. The cherries used in this study are reported to have a concentration of 0–22.4 ng/100 g fresh weight (González‐Gómez et al. 2009), which represented a daily intake of only 0 to 90‐ng melatonin.

Kiwifruit is recognized as a melatonin‐containing food, with an estimated melatonin content of approximately 24,000 ng/g of fruit (Doherty et al. 2023). However, evidence for its sleep‐promoting effects remains inconsistent. While significant improvements in sleep outcomes have been reported in elite athletes (Doherty et al. 2023), other research found no increase in urinary melatonin despite improved sleep onset, suggesting that kiwifruit's sleep‐promoting effects may not be attributable solely to its melatonin content (Kanon et al. 2023). Similarly, subjective improvements in sleep were also observed in another study without actigraphy‐confirmed changes by the consumption of kiwi fruits (Nødtvedt et al. 2017). Together, these findings indicate that the mechanism underlying the sleep‐related effects of kiwifruit remains uncertain. Other bioactive constituents of kiwifruit, including serotonin, antioxidants, vitamin C, and folate, may also contribute to the observed benefits, either independently or through synergistic interactions within the food matrix (Kanon et al. 2023). Therefore, the sleep‐related effects associated with kiwifruit are more likely to reflect the combined action of multiple compounds rather than melatonin alone. Further studies incorporating objective sleep assessments and precise biochemical measurements are needed to better clarify the underlying mechanisms.

### 4.1 Dose–Response Relationship and Optimal Consumption Strategy

The effectiveness of melatonin‐rich foods in improving sleep may depend also on the dose of melatonin consumed, its bioavailability, and the timing of intake. Unlike pharmacological melatonin supplements, which deliver controlled doses in milligrams, dietary sources contain significantly lower concentrations, typically in the microgram or nanogram range. This raises questions about whether food‐derived melatonin is sufficient to induce meaningful physiological effects on sleep. The reviewed studies demonstrated variability in melatonin dosages and consumption patterns, which influenced sleep outcomes. Significant improvements were observed when 85,200 ng/day of melatonin was administrated through tart cherry juice (Howatson et al. 2012), while long‐term supplementation with approximately 500 mg of freeze‐dried tart cherries over 30 days exhibited no measurable effect, suggesting that extended intake may not necessarily enhance sleep benefits in healthy individuals (Hillman et al. 2022). On the other hand, consumption of 250 g of beefsteak tomatoes led to a 10‐fold increase in urinary melatonin, representing one of the most pronounced absorption effects among the studies reviewed (Yang et al. 2020). However, they did not mention the melatonin level of tomatoes in the study.

Although melatonin has been identified in foods across a broad concentration range, the amount delivered through typical dietary interventions is substantially lower than doses used in clinical practice. Melatonin derived from food sources may range from picograms to milligrams per gram depending on the food matrix and source (Zanirate et al. 2026), whereas supplemental melatonin for adults is commonly recommended at doses of 0.2–5 mg administered approximately 60 min before bedtime (Minich et al. 2022). This distinction is important when interpreting the sleep‐related effects of melatonin‐containing foods, as their physiological effects may not be directly comparable to those of pharmacological supplementation. Moreover, these effects may not be explained solely by melatonin content but may also arise from synergistic interactions among other bioactive compounds naturally present in the food matrix, including tryptophan, serotonin, polyphenols, and antioxidant constituents, which may collectively contribute to sleep promotion.

Another limitation is that several studies did not specify the melatonin concentration of the intervention food, making it difficult to determine the clear dose–response relationship of dietary melatonin and its effects on sleep. The lack of this information limits the ability to compare findings across studies and establish optimal dietary intake recommendations. Importantly, many of the existing interventions have not tested the foods with the highest known melatonin concentrations. For example, pistachios (233 ng/g) and walnuts (1.191 ng/g) contain considerably higher melatonin levels than tart cherries (13.5–15.05 ng/g), yet their effects on sleep have not yet been evaluated in interventional studies. Other foods such as grapes, cranberries, goji berries, oats, barley, and mushrooms also contain appreciable melatonin levels but remain under‐researched. Future research should focus on evaluating the sleep‐promoting potential of these richer and more accessible melatonin sources, as they may prove more effective than the foods tested so far.

### 4.2 Age and Population‐Specific Outcomes

Studies on elderly populations (Valtonen et al. 2005; Garrido et al. 2013; Yang et al. 2020) generally reported stronger effects on sleep enhancement compared to those on younger, healthy individuals. The association between melatonin's role in circadian rhythm regulation and age‐related declines in endogenous melatonin production may explain why elderly individuals experienced greater benefits from dietary melatonin interventions. In a study of healthy adults with no reported sleep disturbances, Hillman et al. found that daily consumption of cherry juice did not result in significant improvements in sleep outcomes, suggesting minimal impact when baseline sleep is already stable (Hillman et al. 2022). In contrast, elderly populations and those with sleep disturbances experienced significant improvements, including increased sleep efficiency and reduced sleep latency with Montmorency tart cherry juice (Pigeon et al. 2010). Similarly, other 2 studies in older adults reported substantial benefits on sleep in elderly participants (Losso et al. 2018; Valtonen et al. 2005). Studies on mixed age groups, such as (Garrido et al. 2010, 2013), further support this trend, with notable improvements in sleep efficiency among middle‐aged and elderly participants consuming Jerte Valley cherries. This suggests that melatonin‐rich foods may be most effective in populations with lower natural melatonin levels.

### 4.3 Methodological Heterogeneity and Sources of Variability

Across the included studies, several methodological sources of heterogeneity likely contributed to variability in outcomes and limited direct comparability. Intervention duration differed markedly, ranging from short protocols e.g., 2 days to multi‐week interventions such as 2‐week tart cherry juice trials (Chung et al. 2022; Pigeon et al. 2010). Melatonin exposure (estimated dose) and consumption patterns also varied substantially across interventions, which is important given the circadian regulation of endogenous melatonin and the time‐dependent nature of sleep outcomes. Some studies quantified melatonin content directly within the food matrix (Howatson et al. 2012; Losso et al. 2018), whereas others inferred physiological exposure using biomarkers such as urinary 6‐sulfatoxymelatonin (aMT6s) or serum melatonin (Garrido et al. 2010, 2013; Howatson et al. 2012; Yang et al. 2020; Chung et al. 2022); whereas several studies did not biochemically verify exposure. Additionally, differences in food matrix (e.g., whole food vs. juice vs. concentrate vs. freeze‐dried powder) and processing may alter melatonin stability and absorption, making dose–response relationships difficult to interpret and limiting direct comparisons across studies. Finally, sleep outcomes were assessed using heterogeneous tools subjective measures (sleep diaries, questionnaires, caregiver‐rated scales) and objective methods (actigraphy, EEG devices, and polysomnography) which capture different dimensions of sleep and may partly explain inconsistent findings across food sources and study designs. Taken together, these methodological differences should be considered when interpreting the overall evidence base and may partly explain inconsistent effects across food sources and study designs.

## 5 Strengths and Limitations

A key strength of this scoping review is its systematic and rigorous methodology, conducted in accordance with PRISMA guidelines and supported by a comprehensive literature search across multiple major databases. By synthesizing evidence from both human interventional and observational studies, this review provides an integrated and updated overview of the literature examining the effects of melatonin‐containing foods on sleep‐related parameters in human studies. The methodological quality of included studies was critically appraised using the JBI SUMARI tool, which is appropriate for diverse study designs. Furthermore, the structured screening process, systematic data extraction, and use of validated sleep‐related outcome measures further strengthen the reliability and reproducibility of the findings. This review builds upon and extends existing literature on dietary melatonin intake in humans. While earlier reviews have primarily examined the physiological effects of melatonin‐containing foods, particularly changes in circulating melatonin or related metabolites, the present scoping review extends this work by focusing specifically on functional sleep‐related outcomes, thereby providing clinically relevant insight into the potential role of dietary melatonin in sleep regulation.

Despite these methodological strengths, the following limitations of the review are noted. High heterogeneity was observed in study designs, population characteristics, sleep assessment tools, melatonin dosages, and food consumption methods (juice, whole fruit, or extracts). Several studies focused on individuals with sleep disturbances, while others examined healthy adults, introducing variability in baseline sleep conditions that may have influenced the outcomes. Another limitation of this review is the use of different sleep measurement tools to assess sleep quality. Most studies relied on subjective sleep metrics, and only a few studies incorporated objective measures, which provide more reliable sleep assessments. Additionally, variability in melatonin dosing and timing across different foods and consumption patterns makes it challenging to establish clear dietary recommendations for sleep improvement. Only a few studies mention the melatonin dose present in a particular intervention while the rest of the studies did not mention it. Some studies confirmed increased urinary melatonin levels following the consumption of melatonin‐containing foods, and other studies failed to show significant melatonin absorption, despite reporting sleep benefits. These inconsistencies highlight the complex interplay between melatonin, serotonin metabolism, polyphenols, and other bioactive compounds in sleep regulation. In addition, the RCTs varied significantly in intervention duration, ranging from days to several weeks, which could influence the magnitude and sustainability of melatonin's impact on sleep. Studies with longer intervention periods observed more pronounced and stable improvements in sleep quality compared to shorter trials. This suggests that continuous intake over time may be necessary to achieve significant sleep benefits, particularly in populations with chronic sleep disruptions.

Despite these limitations, the findings suggest that melatonin‐rich foods may have potential as a supportive dietary approach for improving sleep, particularly among individuals experiencing sleep disturbances; however, the evidence remains heterogeneous and not directly comparable to pharmacological interventions. The strongest evidence supports the use of tart cherry juice and Jerte cherries to enhance sleep quality, although the exact mechanisms remain unclear. Along with melatonin, other bioactive compounds such as polyphenols, flavonoids, and tryptophan present in these foods may contribute together to enhance sleep health.

## 6 Conclusion

This scoping review suggests that melatonin‐rich foods may serve as a natural, non‐pharmacological intervention for sleep improvement. However, the effectiveness of any chosen food is influenced by multiple factors beyond the melatonin content of the food, including bioavailability, and individual variability within the food item (e.g., variations in growing conditions that affect nutrient content). Evidence indicates that melatonin‐containing foods may influence sleep through multiple mechanisms, such as serotonin metabolism, antioxidant defense mechanism, and anti‐inflammatory properties. The findings have practical implications for clinicians, dietitians, athletes, and individuals seeking natural sleep‐enhancing strategies. However, current review on melatonin‐containing foods and their effects on sleep remain limited, making it difficult to identify an ideal food for future intervention studies. Furthermore, inconsistencies in study methodologies, including melatonin dosage and timing, and sleep assessment tools, highlight the need for further research to establish recommendations for dietary interventions. Future studies should focus on quantifying melatonin concentrations in different foods available locally to the study participants, optimizing dosing strategies, and integrating biochemical assessments such as serum and urinary melatonin levels to confirm physiological effects. In addition, future interventions should explore a wider range of melatonin‐rich foods such as walnuts, pistachios and other nuts to evaluate their effects on sleep outcomes, as these foods are known to contain higher melatonin levels than some of those currently studied and are also convenient for regular dietary inclusion.

## Author Contributions

**Prajwal Gyawali:** conceptualization, investigation, validation, formal analysis, supervision, writing – review and editing. **Stephen Bird:** supervision, resources, writing – review and editing, validation, formal analysis, conceptualization. **Tanzeela Nisar:** conceptualization, investigation, writing – original draft, methodology, formal analysis. **Andrea Fuller:** conceptualization, writing – review and editing, formal analysis, supervision, software, investigation.

## Funding

The authors have nothing to report.

## Conflicts of Interest

The authors declare no conflicts of interest.

## Supporting information


**TABLE S1:** fsn371823‐sup‐0001‐TableS1.docx.

## Data Availability

The data that support the findings of this study are available from the corresponding author upon reasonable request.
