# Supplementary material for: Effectiveness of Melatonin‐Containing Foods on Promoting Sleep: A Scoping Review
Source: Food Sci Nutr. 2026 Apr 27;14(5):e71823. doi: 10.1002/fsn3.71823 (PMC13121932; doi:10.1002/fsn3.71823)
Supplement: Supplementary file 1 — TABLE S1: fsn371823‐sup‐0001‐TableS1.docx. [file FSN3-14-e71823-s001.docx]

**Supplementary Table S_1_**: search syntax.

| **Database** | **syntax** | **result** |
| --- | --- | --- |
| Pubmed | ((("Diet, Food, and Nutrition"[Mesh]) OR (Diet*[Text Word] OR Food[Text Word] OR Nutrition*[Text Word] OR Natural*[Text Word] OR cherries[Text Word] OR milk[Text Word])) AND (("Melatonin"[Mesh]) OR (Melatonin*[Text Word] OR "phyto-melatonin*"[Text Word] OR Phytomelatonin*[Text Word] OR 6-Sulfatoxymelatonin*[Text Word] ))) AND ((("Dyssomnias"[Mesh]) AND "Sleep"[Mesh]) OR (Sleep*[Text Word] OR Insomnia*[Text Word] OR Dyssomnia*[Text Word] OR Circad*[Text Word] OR “Shift-worker”[Text Word] OR Jetlag*[Text Word])) | 1664 |
| SCOPUS | ( ( ( INDEXTERMS ( "Diet, Food, and Nutrition" ) ) OR ( TITLE-ABS ( diet* ) OR TITLE-ABS ( food ) OR TITLE-ABS ( nutrition* ) OR TITLE-ABS ( natural* ) ) ) AND ( ( INDEXTERMS ( melatonin ) ) OR ( TITLE-ABS ( melatonin* ) OR TITLE-ABS ( melatonin-rich* ) OR TITLE-ABS ( phyto-melatonin* ) OR TITLE-ABS ( phytomelatonin* ) OR TITLE-ABS ( 6-sulfatoxymelatonin* ) ) ) ) AND ( ( ( INDEXTERMS ( sleep ) ) OR INDEXTERMS ( dyssomnias ) ) OR ( TITLE-ABS ( sleep* ) OR TITLE-ABS ( insomnia* ) OR TITLE-ABS ( dyssomnia* ) OR TITLE-ABS ( circad* ) OR TITLE-ABS ( jetlag* ) OR TITLE-ABS ( shift-worker )) ) | 1770 |
| WoS | (((TS="Diet, Food, and Nutrition") OR (TS=Diet* OR TS=Food OR TS=Nutrition* OR TS=Natural* OR TS=milk OR TS=cherries)) AND ((TS=Melatonin) OR (TS=Melatonin* OR TS=phyto-melatonin* OR TS=Phytomelatonin* OR TS=6-Sulfatoxymelatonin*))) AND (((TS=Dyssomnias) AND TS=Sleep) OR (TS=Sleep* OR TS=Insomnia* OR TS=Dyssomnia* OR TS=Circad* OR TS= Jetlag* OR TS=Shift-worker)) | 2119 |
| CINAHL Ultimate | ((((MH "Nutrition+")) OR (Diet* OR Food OR Nutrition* OR Natural* OR milk OR cherries)) AND (((MH Melatonin)) OR (Melatonin* OR phyto-melatonin* OR Phytomelatonin* OR 6-Sulfatoxymelatonin*))) AND ((((MH Dyssomnias+)) AND (MH Sleep+ OR MH "Sleep Disorders+")) OR (Sleep* OR Insomnia* OR Dyssomnia* OR Circad* OR Jetlag* OR shift-worker)) | 445 |
| Total |  | 5998 |
| WoS: web of science. | | |
